# A systematic investigation of the association between network dynamics in the human brain and the state of consciousness

**DOI:** 10.1093/nc/niaa008

**Published:** 2020-06-14

**Authors:** Julia S Crone, Evan S Lutkenhoff, Paul M Vespa, Martin M Monti

**Affiliations:** n1 Department of Psychology, University of California Los Angeles, Los Angeles, CA 90095, USA; n2 Department of Neurosurgery, University of California Los Angeles, Los Angeles, CA 90095, USA

**Keywords:** states of consciousness, fMRI, time-varying network interaction, disorders of consciousness, brain dynamics

## Abstract

An increasing amount of studies suggest that brain dynamics measured with resting-state functional magnetic resonance imaging (fMRI) are related to the state of consciousness. However, the challenge of investigating neuronal correlates of consciousness is the confounding interference between (recovery of) consciousness and behavioral responsiveness. To address this issue, and validate the interpretation of prior work linking brain dynamics and consciousness, we performed a longitudinal fMRI study in patients recovering from coma. Patients were assessed twice, 6 months apart, and assigned to one of two groups. One group included patients who were unconscious at the first assessment but regained consciousness and improved behavioral responsiveness by the second assessment. The other group included patients who were already conscious and improved only behavioral responsiveness. While the two groups were matched in terms of the average increase in behavioral responsiveness, only one group experienced a categorical change in their state of consciousness allowing us to partially dissociate consciousness and behavioral responsiveness. We find the variance in network metrics to be systematically different across states of consciousness, both within and across groups. Specifically, at the first assessment, conscious patients exhibited significantly greater variance in network metrics than unconscious patients, a difference that disappeared once all patients had recovered consciousness. Furthermore, we find a significant increase in dynamics for patients who regained consciousness over time, but not for patients who only improved responsiveness. These findings suggest that changes in brain dynamics are indeed linked to the state of consciousness and not just to a general level of behavioral responsiveness.

## Introduction

The embodiment of the brains’ billions of neurons creates a complex entity across space and time that allows the human mind to be conscious of itself and its environment. Despite major efforts in the past decades to understand neuronal mechanisms underlying processes of consciousness, our understanding of these complex relationships is still elusive. Nevertheless, a large body of work demonstrates that the functional organization of brain regions including frontal and medial posterior areas, the thalamus, and the globus pallidus are altered in impaired states of consciousness in patients (Laureys *et al.* 1999, 2000; Adams *et al.* 2000; Schiff 2008, 2016; Fernandez-Espejo *et al.* 2010, 2012; Lull *et al.* 2010; Vanhaudenhuyse *et al.* 2010; Zhou *et al.* 2011; Crone *et al.* 2014, 2015, 2018; Hannawi *et al.* 2015; Lutkenhoff *et al.* 2015) as well as in anesthetized healthy subjects (Fiset *et al.* 1999; White and Alkire 2003; Greicius *et al.* 2008; Boveroux *et al.* 2010; Deshpande *et al.* 2010; Mhuircheartaigh *et al.* 2010; Guldenmund *et al.* 2013; Ranft *et al.* 2016). It is no coincidence that all of these regions are part of the basal ganglia-thalamo-cortical circuit known to be involved in regulating a critical balance of excitation and inhibition necessary for cortical arousal, motor control, and high-level cognition (Middleton and Strick 2000; Van der Werf *et al.* 2003; Gerardin *et al.* 2004; Nambu 2008; Schiff 2008; Qiu *et al.* 2010; Vetrivelan *et al.* 2010; Bor and Seth 2012; Lazarus *et al.* 2012; Leech and Sharp 2014; Halassa and Kastner 2017; Schmitt *et al.* 2017). Recent approaches, however, shifted their focus from the rather restricted view of the “where” to the more sophisticated “how” focusing on the relation of information to its embodiment rather than locating function based on the notion that the emergence of consciousness is best understood if the complex network interactions are quantified at a spatio-temporal scale (Hoel *et al.* 2013). A previous investigation suggests that the temporal dimension of network connectivity, i.e., alterations in spatial distribution of connectivity patterns over time, specifically represent fluctuations in basic brain states of wakefulness or consciousness (Laumann *et al*. 2018). Indeed, measures of complexity and dynamics have been shown to distinguish states of consciousness in a range of different experimental settings including sleep, anesthesia, and patients with severe brain injury (Massimini *et al.* 2005; Casali *et al.* 2013; Chang *et al.* 2013; Hutchison *et al.* 2013, 2014; Amico *et al.* 2014; Hall *et al.* 2014; Tagliazucchi *et al.* 2014; Tagliazucchi and Laufs 2014; Barttfeld *et al.* 2015; Hudetz *et al.* 2015, 2016; Sarasso *et al.* 2015; Laumann *et al.* 2018; Rosanova* et al.*2018) including a very recent large-sample publication (Demertzi *et al.* 2019). However, in each of these experimental designs, changes in the state of consciousness are also closely associated with changing levels of behavioral responsiveness and cognitive abilities as well as other non-cognitive processes of transition. Independent of the specific metric used to measure brain dynamics, this intertwining association makes it difficult to draw straightforward conclusions regarding the relationship of brain function and consciousness itself, thus limiting the interpretation of these findings. Despite it being common practice to interpret these metrics as revealing changes in states of consciousness, it remains yet to be proven that they are not just a (partial or complete) reflection of changes in behavioral responsiveness.

In this study, we address this issue by adopting an approach that allows to dissociate the state of consciousness from the level of behavioral responsiveness (i.e., the level of residual cognitive function, as well as arousal and motor control as measured with standardized behavioral protocols). Implementing this approach, we assess the dynamics of network properties at the whole brain level and within the basal ganglia-thalamo-cortical loop. We were able to acquire a rare data set of two functional magnetic resonance imaging (fMRI) sessions (acute and follow-up) as part of a longitudinal study in patients who recovered from coma after severe brain injury to a state of wakefulness with either no or only reduced conscious behavior. The patients underwent standardized behavioral evaluation and functional neuroimaging early post-injury (within the first ∼7 days; acute session) and 6 months later (follow-up session). Within this sample, some patients were unconscious at the acute session and, by the follow-up session, had regained both consciousness and behavioral responsiveness (unconscious–conscious group). The other patients, in contrast, could already demonstrate a state of consciousness — if minimal — at the acute session and had improved further behavioral responsiveness by the follow-up session (conscious–conscious group). Crucially, the feature that allows disentangling recovery of consciousness from recovery of behavioral responsiveness is that our sample includes two groups of patients which differ in their (binary/qualitative) state of consciousness (Bayne *et al.* 2016) but not in their (continuous/quantitative) gain of behavioral responsiveness over time.

**Figure 1.**
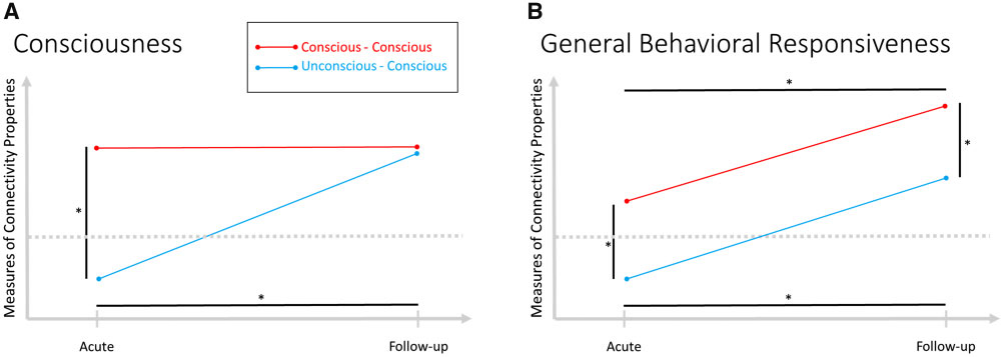
Expected pattern of findings for each hypothesis. (**A**) Expected pattern if consciousness is the driving factor for changes in brain dynamics; significant differences between sessions only for the unconscious-conscious group and between groups only at the acute session. (**B**) Expected pattern if behavioral responsiveness and not consciousness is the driving factor for changes in brain dynamics; *indicates significant differences. Dotted line indicates a change in the state of consciousness.

Given the above, we can make a set of straightforward hypotheses regarding a group of four comparisons, as to which pattern of results would validate previous assumptions that brain dynamics reflect one’s state of consciousness and not just behavioral responsiveness (including implied residual cognitive function). Specifically, if the state of consciousness is the driving factor underlying brain dynamics, we ought to observe (i) a significant difference between the two groups at the acute session (since the state of consciousness differs between the groups) but no significant difference at follow-up (since all patients had recovered consciousness by then), as well as (ii) a significant difference over time for the unconscious–conscious group (since the state of consciousness of these patients changed across sessions) but no significant difference over time for the conscious–conscious group (since the state of consciousness of these patients did not change across sessions) — see pattern A in Fig. 1. In contrast, if brain dynamics are mainly driven by the level of behavioral responsiveness, we expect significant differences for both between assessments and between groups — see pattern B in Fig. 1. In the case that we find significant differences between groups at the follow-up session (since patients in both groups are conscious) or between sessions in the conscious–conscious group (since the state of consciousness did not change), the state of consciousness can be ruled out as the main driving factor underlying changes in time-varying network organization.

## Materials and Methods

### Patient population

Patients were recruited as part of the UCLA Brain Injury Research Center (BIRC). Inclusion criteria were an admission Glasgow Coma Scale (GCS) (Teasdale and Jennett 1974) score of below 8 or an admission GCS of 9–14 with computerized tomography brain scans demonstrating intracranial bleeding, in addition to a stable condition. The main exclusion criteria were a GCS score of above 8 with a non-significant computerized tomography brain scan, history of neurological disease or injury, brain death, and unsuitability to enter the magnetic resonance imaging (MRI) environment (e.g. due to any non MRI-safe implant or medical restrictions). Informed written consent was obtained from the patient’s legal representative. The study was approved by the UCLA institutional review board. For this study, we investigated 55 patients including only patients who were scanned during the acute stage of injury [within 14 days (mean = 7.33) after injury] as well as during the follow-up stage (6 months after injury) and who have recovered consciousness at the follow-up assessment. However, we excluded patients exceeding the threshold for motion (see Motion section) as well as subjects with severe deformation of the head for which we do not expect sufficient registration results. Hence, we excluded 37 of the initial cohort of 55 patients with severe traumatic brain injury resulting in a total of 18 patients [10 male; mean age = 38 years (SD = 17 years); see Supplementary Table S1 for details on each subject]. The unusual high number of exclusions was necessary to obtain a sample of patients with high-quality data in order to perform reliable resting-state analyses (comparable to high-quality data used in experimental studies on healthy subjects).

### Behavioral assessment

Patients were assessed at the day of scanning using the Glasgow Coma Scale (GCS) for the acute session. Since this assessment took place within days of injury, the GCS was the appropriate assessment tool (see Discussion of limitations section). At the follow-up session, the Glasgow Outcome Scale-extended (GOS-e) was performed. It is the standard assessment tool for patients who have recovered from brain injury (see Discussion of limitations section). Based on the GCS subcategories at the acute assessment, we divided the patients into two groups, those who were conscious and those who were not conscious (see Supplementary Table S1) by following a simple rule: If the patient exhibited any sign of voluntary behavior, that is, retrieved one of the following scores on either subscale, Verbal Response of 4 (Confused speech) or 5 (Oriented to time and place) or a Motor Response of 5 (Localization to noxious stimulus) or 6 (Obeys commands), the patient has recovered from unconsciousness as defined by the Coma Recovery Scale-Revised (Giacino *et al*. 2012; Giacino and Kalmar 2006) Although it would have been interesting to include a group of patients who remained unconscious at both time points, we do not have sufficient data for these patients. However, we do believe that this group is not necessary to prove our point since it would not exhibit a dissociation between behavioral responsiveness and level of consciousness.

Only for comparison purposes, the GCS acquired on the day of the acute MRI was transformed into an “inferred GOS-E” score of 2 (if they were diagnosed as vegetative state) or a score of 3 (if they recovered from vegetative state) by using the individual GCS subscale scores. None of the patients had a higher “inferred GOS-E” score than 3 since all patients were in the intensive care unit. With the “inferred GOS-E score,” we then verified that the slope of gain in behavioral responsiveness across time are not significantly different between groups with an ANOVA using aov in R. Although a non-parametric test would have typically been the method of choice for ordinal data, we decided to implement an ANOVA since an ordinal logistic regression cannot handle (i) perfect predictions when one value of a predictor variable is associated with only one value of the response variable; (ii) small sample sizes; and (iii) small cells. All three apply to this dataset.

### MRI data acquisition

For each patient, we acquired one anatomical and one functional MRI data set (among other clinical and research sequences) at each session. Anatomical data were acquired with an MPRAGE sequence (repetition time (TR) = 1900 ms, echo time = 3.52 ms, flip angle = 98). Due to the clinical and highly acute setting, we encounter some variance in the acquisition parameters for the functional data across patients and sessions (see Supplementary Table S2 for details).

### Motion

Due to a high motion likelihood in this patient population, we carefully checked motion parameters to exclude all subjects with motion parameters higher than 2 mm translation and 2 degree of rotation within the whole range of data points based on the acquired voxel size. To do so, we displayed the three translation and three rotation parameters across the whole run and identified time-series with peaks above the threshold. We also excluded all subjects with framewise displacement above 0.5 mm using FSL_motion_outliers in the FMRIB Software Library (FSL) as suggested by Power *et al.* (2012).

Later in the processing pipeline, we used the three translation and three rotation parameters, their temporal difference, square, and square of the differenced values identified with FSL MCFLIRT (Jenkinson *et al.* 2002), as well as the aCompCor results for white matter and cerebrospinal fluid (see below) using mp_diffpow.sh as nuisance regressors to linearly regress out motion. We decided to implement a regression approach instead of censoring techniques as a preferred method to cope with motion-related artifacts (Power *et al.* 2014). The main reason why we did not use censoring is because this would interrupt the continuous time-series (especially problematic when investigating dynamics of connectivity), lead to unequal number of time points across subjects, and substituting this fact by means of replacing the particular time points may have broad effects on identifying variance or similar states of covariance across time (Power *et al.* 2015). Another major limitation is that censoring does not correct for subtler influences of motion that do not meet the arbitrary threshold of data to be censored. Instead, to define nuisance regressors, we have implemented an approach using principal component analysis on well-defined masks of white matter and cerebrospinal fluid to define nuisance regressors (referred to as anatomical CompCor or aCompCor). This approach has shown to outperform other approaches for resting-state analyses in general (Muschelli *et al.* 2014; Power *et al.* 2018) and for dynamic functional network connectivity in particular (Vergara *et al.* 2017). As suggested by Muschelli *et al.* (2014) as the optimal solution, we included the number of principal components needed to explain 50% of the variance in white matter and 50% of the variance in cerebrospinal fluid (referred to as aCompCor50). Furthermore, it has been recently shown that this strategy, when paired with stringent data selection, is as effective as censoring (Parkes *et al.* 2018). In addition, we have also implemented an independent component analyses (ICA) to functionally parcellate the brain which, as a side effect, extracts a signal that is to a large extent free of noise and artefacts (Power *et al.* 2015).

### Data preprocessing

Due to the high deformation of brains and severe lesions in patients with traumatic brain injury, we scull-stripped the structural data using an optimized algorithm specifically suitable for this population as implemented in optiBET (Lutkenhoff *et al.* 2014). Structural images were deobliqued using 3drefit with the option *-deoblique* to remove oblique information from the image header and reoriented into right-to-left posterior-to-anterior inferior-to-superior (RPI) orientation using analysis of functional neuroImages (AFNI) 3dresample with the option *-orient RPI*. Then, the structural files were segmented into white matter, gray matter, and cerebrospinal fluid using FSL FAST. Success of segmentation results were verified visually for each image.

Our choice of preprocessing steps is roughly based on the scripts of the 1000 Functional Connectomes Project (http://fcon_1000.projects.nitrc.org) using FSL (Jenkinson *et al.* 2012) and AFNI (Cox 1996). Details are outlined below.

First, resting-state fMRI data of all subjects were trimmed using fslroi discarding the first 4 time points to allow for magnetization stabilization. Second, we performed slice-time correction to account for different acquisition timing using FSL slicetimer specifying TR of data (option *-r*) and acquisition order (option *–odd*). Third, we deobliqued the slice-time corrected data to remove oblique information from the image header with AFNI 3drefit with the option *-deoblique*. In a next step, we reoriented the images using 3dresample with the option *-orient RPI*. Next, we generated the motion parameters to use as nuisance regressors in subsequent analyses for motion correction (see Motion section) using FSL mcflirt (Jenkinson *et al.* 2002) and mp_diffpow.sh. We then created a brain-only mask by detecting the edges of the brain and removing the skull using AFNI 3dAutomask dilating the mask outwards once (option *-dilate 1*). Then, we applied this mask to the reoriented data with AFNI 3dcalc using the expression *-expr* “a*b” to zero out everything outside of the brain. We then calculated and skull-stripped the mean image of each subject using AFNI 3dTstat option *-mean*. Then smoothing was performed with a 5 mm smoothing kernel using *fslmaths –kernel gauss –fmean*. To eliminate any global differences across subjects, we grand mean scaled the smoothed data with fslmaths –ing 10000 –odt float.

### Registration

We did not register the individual subject’s images into standard Montreal Neurological Institute (MNI) space since in patients with lesions and deformations this procedure is highly prone to error. Thus, we decided to create a study-specific average template instead to register all subjects into the same space. To do so, we performed a six-step approach: (i) we randomly picked the structural image of one subject as a reference image and registered the structural images of all other subjects to the selected image using FSL FLIRT *–dof 12*; (ii) we calculated the average image from all the registered images including the reference image using FSL fslmaths with the *–add* and the *–div* (total number of subjects) as well as the *–odt float* option; (iii) We then registered the original functional images from all subjects to their structural images using FSL FLIRT *–dof 6*; (iv) all original structural images were registered to the average template using FSL FLIRT; (v) we then concatenated the mat files using convert_xfm; (vi) then the functional images were registered into the average template space using FSL FLIRT. A linear transformation using FLIRT was chosen since it resulted in the best outcomes and the fewest loss of subjects due to processing failure in comparison with non-linear methods such as FSL FNIRT and ANTSapplytransforms (data not shown).

### Brain parcellation using independent component analysis

**Figure 2.**
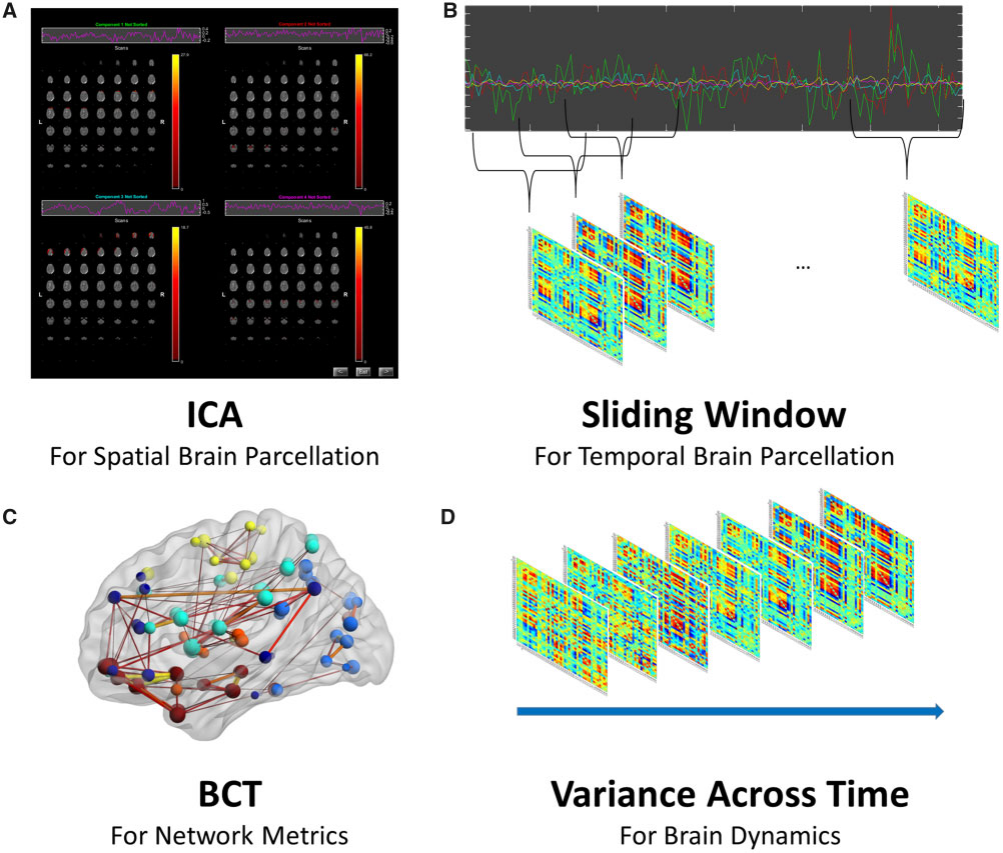
Data processing steps to analyze time-varying network dynamics. (**A**) Spatial group independent component analysis (ICA) using 100 components to define the nodes of the graphs; time course of the 43 remaining independent component networks were further processed including detrending, multiple regression of 18 realignment parameters, and a high-frequency cutoff at 0.15 Hz. (**B**) Sliding-window approach (width = 20 TR) with a 43 × 43 correlation matrix for each of the windows. (**C**) Network metrics for each window included node strength and clustering coefficient using the brain connectivity toolbox. (**D**) To investigate time-varying network organization, the variance across time is assessed for each metric.

A graphical presentation of the processing steps and data analyses to investigate time-varying network properties is shown in Fig. 2. First, we performed ICA using the GIFT toolbox (http://mialab.mrn.org/software/gift/index.html) to parcellate the brain into components, i.e., regions of interest as implemented in Allen *et al.* (2014). We have chosen this form of parcellation (also see our previous publications, e.g., Crone *et al.* 2015) as opposed to anatomical-based or functional-based standard atlases, because the brains we are investigating are severely damaged. The normalization procedure warping the severely damaged brain into standard MNI space is, as mentioned above, a suboptimal solution and should be avoided if possible. It is also problematic to assume that the anatomical or functional parcellation resulting from the average of young and healthy brains adequately represents the functional partitioning that can be found in a brain that has been disrupted and subject to reorganization due to traumatic brain injury. Thus, we defined the regions of interest at an individual level based on the individual functional covariance using group ICA. We ran ICA with 100 components using ICASSO randinit with 10 runs, the Infomax algorithm, and a PCA Expectation Maximization with stacked data sets, a floating point precision, 1000 numbers of iterations, and two reduction steps. The data have been intensity normalized and scaled to percent signal change. Then, the components were visually inspected to exclude all components of noise and artifacts resulting in 43 components/regions of interest (see Supplementary Fig. S1 for all components included).

### Assessing time-varying network properties using a sliding time window approach

Since brain function is not stationary and recent work has found growing evidence for a significant association between brain dynamics and the state of consciousness, we used time-varying network properties as our brain measure of interest (please see Discussion section for a thorough debate on brain dynamics). For reasons of comparability and because there is yet no consensus on which is the best approach to investigate temporal dynamics (Preti *et al.* 2017; Lurie *et al.* 2019), we implemented the same sliding-window approach as described in Barttfeld *et al.* (2015) and in Allen *et al.* (2014) using the dynamic functional network connectivity (dFNC) toolbox integrated in GIFT (http://mialab.mrn.org/software/gift/index.html). We specified all 43 selected components to be included in further analyses and sorted these regions of interest according to the network they belong to, based on a similar organization scheme as presented in Allen *et al.* (2014). As described in the Motion section above, we used the six motion parameters, their temporal difference, square, and square of the differenced values, as well as the outliers as covariates to be regressed out from each time course. In addition, we also used age as a covariate. Using a sliding window approach, covariance matrices were computed from windowed segments of the time-course of the regions of interest as computed in the previous step. We used a tapered window with a size of 20 TRs (Leonardi and Van De Ville 2015) with a Gaussian (*σ* = 3 TRs) and a slide in steps on 1 TR (number of repetition = 10) resulting in 185 windows. Previous studies have shown that windows between 30 and 60 s are best suited to capture fluctuations in resting-state data and that different window lengths (when chosen within this limit) do not result in different findings (Preti *et al.* 2017). To promote sparsity and address the problem of a limited window size (Xu and Lindquist 2015), we estimated covariance from the regularized precision matrix placing a penalty on the L1 norm with 10 numbers of repetition. We despiked and detrended (option 3) the data using a high-frequency cutoff at 0.15 Hz. In addition, we specified the TR for each data set separately.

### Network metrics

We focused on two network metrics, node strength and clustering coefficient (see Discussion section for the rationale of choice). Node strength is a measure of centrality at the local level quantifying the influence, and thus, the capability for information integration of every node within the network. Clustering coefficient is a higher order term measuring the degree to which nodes and their nearest neighbors tend to cluster together revealing therefore the amount of segregation. To calculate each metric, we first normalized the correlation matrix (*r*) using the algorithm, weight_conversion (W, “normalize”), provided by the Brain Connectivity Toolbox (Rubinov and Sporns 2010). We calculated node strength (strengths_und_sign.m) and clustering coefficient (clustering_coef_wu_sign.m) with the Brain Connectivity Toolbox (Rubinov and Sporns 2010) for the whole brain as well as for our four specific regions within the basal ganglia-thalamo-cortical circuit, i.e., medial frontal cortex, posterior cingulate cortex, thalamus, and globus pallidus. For the frontal regions of interest, we took the mean of all included components representing medial frontal areas. For the posterior cingulate cortex, thalamus, and the globus pallidus, we identified the component corresponding the most with our region of interest (see Supplementary Fig. S1). For further analyses, we focused on the metrics for the positive correlations only, since the interpretation of positive correlations is straightforward in contrast to negative correlations. Instead of investigating differences between brain states, we used the summary statistic variability for the assessed metrics because previous work suggests that identification of states is less reliable and may be sensitive to features of data acquisition that vary between subjects (Choe *et al.* 2017). Because of the severe injury in patients’ brains, we expect especially in our cohort to find more variation between subjects than between groups.

### Testing patterns in the data

In this study, we implemented different approaches to test the pattern of data: (i) non-parametric frequentist testing, (ii) permutation frequentist testing, (iii) effect sizes, and (iv) Bayesian testing. Because of the low number of subjects per cell, non-normal data, and unequal variances, we used non-parametric tests in JASP (JASP Team (2019); Version 0.11. 1), i.e., the Mann–Whitney or the Welch test for independent data and the Wilcoxon for paired data. To address the above mentioned issues (i.e. especially the small sample size), we also verified our results with permutation t-tests in R (paired.perm.test in Broman library and oneway_test in Coin library). All *P*-values were corrected for multiple comparisons for the different metrics and regions using false discovery rate (FDR) (Benjamini and Hochberg 1995) to increase power due to our small sample size. Please note that we did not correct for the multiple tests between cells because we are not interpreting the results of each test but the overall pattern. Given the relatively small sample size of the analyzed cohort, we also included a parallel effect size analysis in JASP in order to estimate the size of all significant effects. Finally, to be able to confirm our pattern (rejection of the null hypothesis for the acute session and for the unconscious–conscious group as well as confirmation of the null hypothesis for the follow-up session and the conscious–conscious group), we additionally used Bayesian statistics in JASP for all cases that demonstrate the pattern of interest. Bayesian analysis allows to evaluate which of the hypotheses (alternative or null) are more likely given the data, and thus, provides evidence *for* or *against* a specific hypothesis. We ran Bayesian independent (Mann–Whitney) and paired t-tests (since there is no Bayesian non-parametric option for paired data in JASP) for each comparison, respectively, calculating the Bayes factor to quantify evidence either for the alternative hypothesis (for comparisons between groups at the acute session and within groups for the unconscious–conscious group) or for the null hypothesis (for comparisons between groups at the follow-up session and within groups for the unconscious–conscious group). We used the default prior Cauchy with a scale of 0.707 (Wagenmakers *et al.* 2018). Incorporating the prior knowledge resulting from the frequentist testing, we specified the direction of the test for all comparisons. We also applied a robustness analysis in cases in which it was applicable (paired data) to quantify the evidential impact of the width of *r* for the Cauchy prior distribution (Wagenmakers *et al.* 2018).

### Low-level behavioral responsiveness versus high-level behavioral responsiveness

To further assess the relationship between brain dynamics and behavioral responsiveness, we also compare brain dynamics between two groups of patients which, despite including both conscious patients, demonstrate very different levels of behavioral responsiveness. Based on a previously introduced taxonomy (Bruno *et al.* 2011), we subcategorize the conscious patients (for the acute session only) by distinguishing those who exhibit “high-level behavioral responses (i.e. command following, intelligible verbalizations or non-functional communication),” referred to as minimally conscious state “plus” (MCS+), from those who exhibit “low-level behavioral responses (i.e. visual pursuit, localization of noxious stimulation or contingent behavior such as appropriate smiling or crying to emotional stimuli),” referred to as MCS “minus” (MCS−). We then separated the patients into two groups including only MCS+ in the high-level behavioral responsiveness group and the others in the low-level responsiveness group. If brain dynamics are indeed a reflection of the state of consciousness and not of behavioral responsiveness (and implied cognitive functioning), despite the very different behavioral profile of the two patient groups, they ought not to differ significantly in their brain dynamics. The same analyses were applied as described in the paragraph above.

### Stationary network metrics

Finally, we also compare the network dynamics results obtained with the sliding-window approach described above to the results obtained from a more conventional “stationary” approach—i.e. an approach in which each network metric is calculated over the full length of the time-series (as opposed to being calculated over each of the 185 windows obtained from each time-series). Consequently, in the stationary approach—which can be thought of as a windowed approach with only one window encompassing the full time-series—the dependent variable is the value of each network metric itself rather than the variance of network metrics across 185 windows. All other aspects of the analysis (e.g. calculation of network metrics and statistical comparisons) were carried out as in the dynamics analysis described above.

## Results

### Behavioral assessment

**Figure 3.**
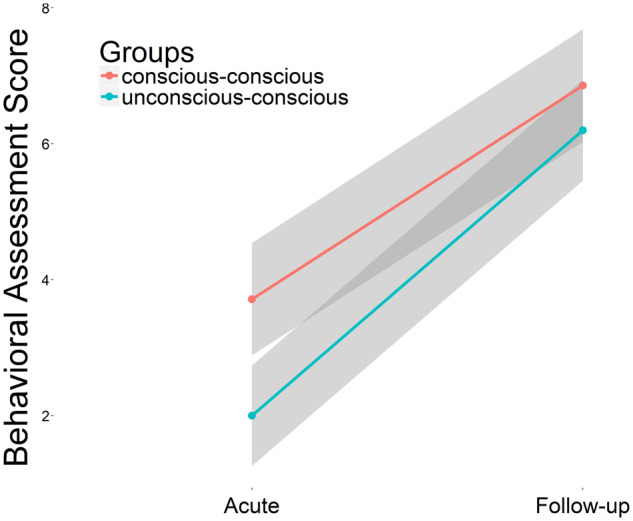
Gain in level of responsiveness of both patient groups. Means and confidence intervals for behavioral responsiveness of both groups (unconscious-conscious and conscious-conscious) is shown for both sessions as assessed with the inferred GOS-E at the acute session and the GOS-E at the follow-up session.

According to the behavioral assessment using the GCS (Teasdale and Jennett 1974) and the GOS-E (Jennett *et al.* 1981) at the acute and follow-up session, respectively, we divided the patients into the unconscious–conscious group (*N* = 10) and the conscious–conscious group (*N* = 8). The ANOVA testing the effects on general behavioral responsiveness as measured by the total score revealed a significant effect for group (*F *=* *4.398, *P *=* *0.044), for session (*F *=* *69.123, *P *<* *0.001), and no interaction effect (*F *=* *0.629, *P *=* *0.434) (see Fig. 3). These findings confirm the dissociation of the state of consciousness and the level of behavioral responsiveness.

### Dynamic functional connectivity

**Figure 4.**
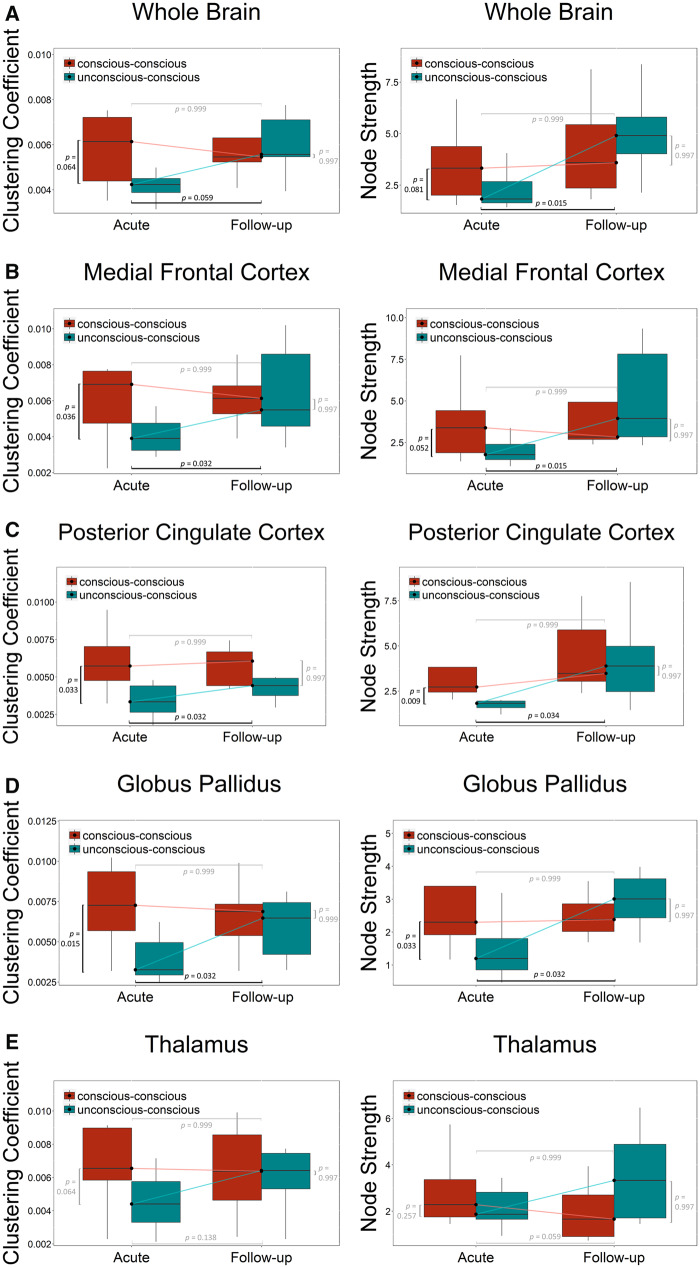
Pattern of differences in time-varying network interaction of clustering coefficient and node strength. Box plots for the whole brain (**A**), the medial frontal cortex (**B**), the posterior cingulate cortex (**C**), the globus pallidus (**D**), and the thalamus (**E**) displaying the median, maximum and minimum value, as well as the quartiles. FDR-corrected *P*-values are displayed at the top for the conscious–conscious group, at the bottom for the unconscious–conscious group, on the left side for the acute session and on the right side for the follow-up session. Black bars indicate a difference between groups, gray bars no difference.

As shown in Fig. 4 and Table 1, the pattern of differences we observed across groups and time points conforms exactly to the expected “consciousness pattern” (cf. Fig. 1A) at the whole brain level, for medial frontal cortex, posterior cingulate cortex, and globus pallidus, but not for thalamus.

**Table 1.** Main results for the dynamic network interaction analyses.

Brain area	Comparison	*p*	*p*_corr_	*r*	Effect size	BF_1_	BF_0_
Whole brain clustering coefficient	Acute between groups	0.051	0.064	0.475	Medium	1.511	0.662
	Unconscious–conscious within group	0.053	0.059	0.6	Large	1.739	0.575
	Follow-up between groups	0.829	0.997	0.075	Negligible	0.415	2.408
	Conscious–conscious within group	0.999	0.999	0	Negligible	0.391	2.56
Whole brain node strength	Acute between groups	0.073	0.081	0.425	Medium	1.534	0.652
	Unconscious–conscious within group	0.003	0.015	0.927	Large	23.77	0.042
	Follow-up between groups	0.408	0.997	0.250	Small	0.498	2.008
	Conscious–conscious within group	0.547	0.999	0.278	Small	0.477	2.098
MFC clustering coefficient	Acute between groups	0.018*	0.036*	1.209*	Large	2.235	0.447
	Unconscious–conscious within group	0.019	0.032	0.745	Large	5.356	0.187
	Follow-up between groups	0.696	0.997	0.125	Small	0.453	2.208
	Conscious–conscious within group	0.742	0.999	0.167	Small	0.447	2.237
MFC node strength	Acute between groups	0.031	0.052	0.425	Medium	1.823	0.548
	Unconscious–conscious within group	0.003	0.015	0.927	Large	17.0	0.059
	Follow-up between groups	0.573	0.997	0.175	Small	0.443	2.309
	Conscious–conscious within group	0.383	0.999	0.389	Medium	0.406	2.463
PCC clustering coefficient	Acute between groups	0.01	0.033	0.65	Large	4.038	0.248
	Unconscious–conscious within group	0.019	0.032	0.745	Large	4.376	0.229
	Follow-up between groups	0.203	0.997	0.375	Medium	0.711	1.407
	Conscious–conscious within group	0.844	0.999	0.111	Small	0.355	2.814
PCC node strength	Acute between groups	0.0009	0.009	0.85	Large	10.301	0.097
	Unconscious–conscious within group	0.024	0.034	0.709	Large	4.835	0.207
	Follow-up between groups	0.897	0.997	0.05	Negligible	0.45	2.22
	Conscious–conscious within group	0.148	0.999	0.611	Large	0.742	1.347
GP clustering coefficient	Acute between groups	0.003	0.015	0.75	Large	7.41	0.135
	Unconscious–conscious within group	0.01	0.032	0.818	Large	2.092	0.478
	Follow-up between groups	0.999	0.999	0	Negligible	0.419	2.384
	Conscious–conscious within group	0.547	0.999	0.278	Small	0.506	1.975
GP node strength	Acute between groups	0.013	0.033	0.625	Large	3.88	0.258
	Unconscious-conscious within group	0.014	0.032	0.782	Large	2.549	0.392
	Follow-up between groups	0.315	0.997	0.3	Medium	0.532	1.879
	Conscious-conscious within group	0.945	0.999	0.056	Negligible	0.407	2.457
Thalamus clustering coefficient	Acute between groups	0.051	0.064	0.475	Medium	1.339	0.747
	Unconscious–conscious within group	0.138	0.138	0.418	Medium	0.876	1.141
	Follow-up between groups	0.762	0.997	0.1	Small	0.484	2.066
	Conscious–conscious within group	0.742	0.999	0.167	Small	0.432	2.313
Thalamus node strength	Acute between groups	0.257	0.257	0.2	Small	0.693	1.442
	Unconscious–conscious within group	0.053	0.059	0.6	Large	1.717	0.582
	Follow-up between groups	0.237	0.997	0.35	Medium	0.636	1.571
	Conscious–conscious within group	0.547	0.999	0.278	Small	0.364	2.746

*P* represents the uncorrected *P*-value for the Mann–Whitney (independent data) and Wilcoxon test (paired data), respectively; *p*_corr_ represents the FDR corrected *P*-value; *r* represents the rank biserial correlation coefficient for the effect size; BF_1_ represents the Bayes factor for the alternative hypothesis; BF_0_ represents the Bayes factor for the null hypothesis; GP, globus pallidus; PCC, posterior cingulate cortex; MFC, medial frontal cortex. *indicates that the Welch test and Cohen’s *d* for effect size has been used due to unequal variances.

At the whole brain level (Fig. 4A), we observe a trending difference between groups at the acute session (*P *=* *0.064 for the clustering coefficient and *P *=* *0.81 for the node strength) but not at the follow-up session (*P *=* *0.997 for the clustering coefficient and *P *=* *0.997 for the node strength). Between sessions, we found a significant difference in the unconscious–conscious group (*P *=* *0.015 for the node strength, and a trend, *P *=* *0.059, for the clustering coefficient) but not for the conscious–conscious group (*P *=* *0.999 for the clustering coefficient and *P *=* *0.999 for the node strength).

The same pattern was observed for the medial frontal cortex (Fig. 4B). We see a significant difference between groups at the acute session (*P *=* *0.036 for the clustering coefficient and a trend, *P *=* *0.052 for the node strength) but not at the follow-up session (*P *=* *0.997 for the clustering coefficient and *P *=* *0.997 for the node strength). Between sessions, we found a significant difference in the unconscious–conscious group (*P *=* *0.032 for the clustering coefficient and *P *=* *0.015 for the node strength) but not for the conscious–conscious group (*P *=* *0.999 for the clustering coefficient and *P *=* *0.999 for the node strength).

The posterior cingulate cortex (Fig. 4C) also showed significant differences between groups at the acute session (*P *=* *0.033 for the clustering coefficient and *P *=* *0.009 for the node strength) but none at the follow-up session (*P *=* *0.997 for the clustering coefficient and *P *=* *0.997 for the node strength). We also found a significant difference between sessions in the unconscious–conscious group (*P *=* *0.032 for the clustering coefficient and *P *=* *0.034 for the node strength) but no differences between sessions for the conscious-conscious group (*P *=* *0.999 for the clustering coefficient and *P *=* *0.999 for the node strength).

In the globus pallidus (Fig. 4D), we found a significant difference between groups at the acute session (*P *=* *0.015 for the clustering coefficient and *P *=* *0.033 for the node strength) but not at the follow-up session (*P *=* *0.997 for the clustering coefficient and *P *=* *0.997 for the node strength). Between sessions, we found a significant difference in the unconscious–conscious group (*P *=* *0.032 for the clustering coefficient and *P *=* *0.032 for the node strength) but not for the conscious–conscious group (*P *=* *0.999 for the clustering coefficient and *P *=* *0.999 for the node strength).

Interestingly, the thalamus did not show the “consciousness pattern” (Fig. 4E). There was no significant difference between groups neither at the acute session (a trend, *P *=* *0.064, for the clustering coefficient and *P *=* *0.257 for the node strength) nor at the follow-up session (*P *=* *0.997 for the clustering coefficient and *P *=* *0.997 for the node strength). Between sessions, there was also no significant difference in the unconscious–conscious group (*P *=* *0.138 for the clustering coefficient and a trend, *P *=* *0.059 for the node strength) nor in the conscious–conscious group (*P *=* *0.999 for the clustering coefficient and *P *=* *0.999 for the node strength). All *P*-values reported above are FDR corrected.

As expected, the permutation t-tests confirm the non-parametric findings (see Supplementary Table S3).

The effect size analysis also confirms the pattern found in the frequentist analyses with all significant comparisons between groups and sessions demonstrating a high effect with at least 0.625 and at least 0.425 for all trending comparisons (see Table 1).

The Bayesian analysis also supports the general pattern (see Supplementary Fig. S2). In most cases in which the alternative hypothesis (H_1_) is favored, we find moderate (in some cases even strong) evidence demonstrating that the alternative hypothesis is at least 3× or more likely than the null hypothesis (H_0_). The robustness analysis confirms moderate evidence across different widths *r* (see Supplementary Fig. S3). In cases in which the null hypothesis (H_0_) is favored, evidence is between anecdotal and moderate meaning that the null hypothesis is at least around 2× more likely. The robustness analysis demonstrates that as the width *r* increases, the Bayes factor increases as well ranging between anecdotal and moderate (see Supplementary Fig. S3).

### Low-level behavioral responsiveness versus high-level behavioral responsiveness

Consistent with the above reported results, when we compared brain dynamics for patients differing only in their level of behavioral responsiveness (i.e. high-level versus low-level), we failed to observe any significant difference between groups or between sessions. Supplementary Fig. S4A–E shows the results from the non-parametric testing and Supplementary Table S3 the results from the permutation t-tests.

### Stationary functional connectivity

Intriguingly, when testing for differences in metrics for stationary functional connectivity in contrast to dynamic functional connectivity, we also could not detect any differences between groups at either session nor between sessions in either group (see Supplementary Fig. S5A–E for results from the non-parametric t-tests and Supplementary Table S3 for the results from the permutation t-tests).

The results for the effect size confirm the findings of the frequentist analyses failing to reveal the pattern of interest (see Supplementary Table S4).

## Discussion

This study was designed to address the question of whether brain dynamics are best interpreted as neuronal correlates of the state of consciousness (as so far only assumed in previous studies) or whether they rather reflect the level of behavioral responsiveness (and implied cognitive functioning). For example, a recent study in a large sample of patients and anesthetized healthy volunteers has identified a complex dynamic pattern of coordinated and anti-coordinated fMRI signals that characterizes conscious subjects in contrast to unconscious subjects (Demertzi *et al.* 2019). However, the reported differences in the state of consciousness are confounded by a simultaneous improvement of behavioral responsiveness as it is the case in most studies in this field. Despite including a clever comparison across two patient groups with different states of consciousness (as revealed by functional MRI) but matching assessment of behavioral responsiveness (as revealed by bedside clinical assessment), the comparison is still limited by the same confounding factor. Although the two patient groups appear behaviorally matched, the fact that patients in one group could voluntarily engage in a complex mental activity (e.g. “imagine playing tennis”) indicates a state of cognitive-motor dissociation (Demertzi *et al.* 2019). Given the high cognitive demand of mental imagery even for healthy subjects, patients successfully performing such a task must exhibit significant residual cognitive functioning, e.g., sufficient working memory, language comprehension, and attention span (Pearson *et al.* 2015). Consequently, the higher probability of a complex dynamic pattern in patients with cognitive-motor dissociation can still be attributed to differences in residual cognitive functioning as opposed to differences in the state of consciousness.

The present study is the first to specifically address this conflation by dissociating differences in the state of consciousness from differences in behavioral responsiveness. Even though a precise dissociation is not possible due to the non-linear properties of recovery in cognitive function (please see the Limitation section for a detailed discussion of this problem), our results provide very strong evidence supporting previous claims that brain dynamics are mainly associated with the state of consciousness as opposed to the level of behavioral responsiveness (and implied residual cognitive functioning). The results match exactly the four-pronged pattern we expected to observe if indeed the state of consciousness is the driving force of time-varying network properties (compare Fig. 1A to Fig. 4A–D). While we found significant differences, both within and between patient groups, when there was a qualitative change in state of consciousness, we could not detect any such difference when there was a quantitative change in the level of behavioral responsiveness but not in consciousness. This pattern was observed at the overall whole-brain level as well as in key sub-regions selected on the basis of prior literature (Schiff 2010) and confirmed by a set of multiple analysis strategies including non-parametric and permutation frequentist statistics as well as Bayesian statistics. In contrast, none of the analyses revealed the “general behavioral responsiveness” pattern (see Fig. 1B). To further confirm the pivotal role of the state of consciousness in the observed pattern (in contrast to behavioral responsiveness), we divided the patients in two groups based on their level of behavioral responsiveness rather than the state of consciousness. Consistent with the above results, the groups do not differ in their dynamic network metrics despite differences in behavioral responsiveness which provides further evidence that brain dynamics best reflect the state of consciousness in contrast to the level of behavioral responsiveness.

Interestingly, the thalamus was the only tested region that did not follow this pattern. These results are especially intriguing considering previous findings suggesting a reduced role of the thalamus in recovery of the state of consciousness (Crone *et al.* 2017, 2018). However, it is possible that a more subtle effect might be observable with a larger sample, even though the effect size analysis confirms that changes in time-varying network properties are related to changes in the state of consciousness in all regions that show significant results.

The present results also touch upon the ongoing debate whether frontal or posterior regions are the “hot spot” of consciousness (Boly *et al.* 2017; Dienes *et al.* 2017). While the present design was not designed to test the sufficiency of either brain regions for the emergence of consciousness, our findings do indicate that both seem to play a critical role for dynamic network organization underlying the state of consciousness. We would also like to emphasize in this context that the present study focuses on brain function underlying the *state* of consciousness as opposed to conscious processing of stimuli and subjective conscious experience (Dehaene and Changeux 2011) since these two concepts should be clearly distinguished regarding the ongoing debate.

In addition, we also report that a stationary representation of functional connectivity properties, in contrast to its dynamic representation, does not sufficiently reflect the state of consciousness. In fact, when adopting a stationary, time-invariant approach to estimating properties of neural functional connectivity, we could not detect any significant difference across groups and between sessions. While this finding is interesting, and tackles an important methodological question, a general investigation of brain dynamics goes beyond the scope of this study and specific research is needed focusing on adequate methodological approaches to provide deeper insight into this issue.

Consistent with prior studies, we also find that the variability in the variance of functional connectivity properties from subject to subject is lowest in the group of unconscious patients and highest for the group of patients that are conscious.

Finally, interpretation of these findings should be mindful of a number of limitations. First, brain dynamics in fMRI resting state have been a subject of debate regarding their neuronal origin, methodological implementation, and reliability (Abrol *et al.* 2017; Lehmann *et al.* 2017; Liegeois *et al.* 2017; Preti *et al.* 2017; Kucyi *et al.* 2018; Laumann *et al.* 2018; Miller *et al.* 2018; Thompson 2018; Lurie *et al.* 2019). Although there is a need for more thorough investigations in the future (Preti *et al.* 2017; Lurie *et al.* 2019), a large body of work has already demonstrated a neurophysiological basis of brain dynamics in fMRI data (de Pasquale *et al.* 2010; Tagliazucchi *et al.* 2012; Thompson *et al.* 2013) as well as its reproducibility (Abrol *et al.* 2017; Choe *et al.* 2017; Vidaurre *et al.* 2018). Moreover, most criticism regarding whether brain dynamics are biological meaningful is directed towards their role as a correlate of cognitive processing itself and content of cognition (Laumann *et al.* 2018, see also Discussion in Lurie *et al.* 2019). With respect to the state of arousal and consciousness, there seems to be much more consensus on the fact that brain dynamics may indeed be reflective of these fundamental state changes. Even though part of this complex activity remains during total loss of consciousness (Keilholz *et al.* 2013), significant differences have been demonstrated between different states (Chang *et al.* 2013; Hutchison *et al.* 2013, 2014; Amico *et al.* 2014; Hall *et al.* 2014; Tagliazucchi *et al.* 2014; Tagliazucchi and Laufs 2014; Barttfeld *et al.* 2015; Hudetz *et al.* 2015; Laumann *et al.* 2018; Demertzi *et al.* 2019).

Second, one should be mindful of the fact that recovery of consciousness and responsiveness is not a linear process and that the linear relationship between these two quite distant time points as indicated by the straight lines in Fig. 1, although necessary for this approach, is most certainly an oversimplification; and although the quantitative measure of the gain of behavioral responsiveness did not significantly differ between groups, there might be qualitative differences which we could not observe. Differences in quality, as demonstrated by the ongoing debate concerning accurate diagnoses in patients with disorders of consciousness (e.g. Childs *et al.* 1993; Andrews *et al.* 1996; Owen *et al.* 2006; Schnakers *et al.* 2009), are difficult to assess and compare. The findings of this study rely on the assumption that the absolute changes in the level of responsiveness are comparable, and thus, follow a linear trend at either end of the scale. However, the reality is probably a much more complex, non-linear development, especially regarding the recovery of cognitive function. We know little about the trajectory of changes in cognitive function during recovery from severe brain injury. Most likely, changes in cognitive function at the lower end of the scale are of critical importance and not directly comparable to changes at the higher end of the scale in a quantitative sense. Thus, these non-linear changes are still an issue in the present study and may confound our results. In this sense, the conclusions of this study should be considered with a grain of salt and in the context of the limitations intrinsic to the experimental study dissociating consciousness from other aspects of behavioral responsiveness in patients who do not have the ability to express themselves (Monti and Owen 2010).

Third, to verify the four-pronged pattern we expected to observe if the state of consciousness is the driving force of time-varying network properties, we implemented additional Bayesian hypothesis testing which allows to specifically quantify evidence in favor of either hypothesis in contrast to frequentist statistics which are exclusively designed to quantify evidence against a pre-specified null hypothesis that is sought to be rejected. However, when testing how likely the null hypothesis is given the data, the Bayes factor is quite low in most cases and reaches only anecdotal to moderate evidence depending on the width of the prior. To deal with the very small numbers of subjects per cell, we have additionally implemented permutation testing for the frequentist statistics. However, JASP does not provide an equivalent Bayesian approach. A very small Bayes factor (close to 1) may suggest a not sufficient amount of data for the analyses (Dienes 2014). This may also play into account explaining the weak evidence of the Bayesian approach. Altogether though, the Bayesian analyses confirms the general pattern that all our analyses reveal supporting the conclusion that time-varying network properties depend rather on the state of consciousness than on the general level of responsiveness.

Fourth, severe brain injury patients are often prone to high rates of in-scanner motion and wide-spread lesions. Given the known deleterious effects of motion and distortions on network metrics, we have excluded patients with higher motion and severe lesions. While the above strategy resulted in loss of a large part of our original sample, it does ensure that the data are of high quality in this patient population (similar to what is standard in experimental studies using high-quality healthy-subject data). Nevertheless, in-scanner motion is a critical issue for studies investigating functional connectivity (Power *et al.* 2012; Satterthwaite *et al.* 2012; Van Dijk* et al.* 2012) including brain dynamics (Laumann *et al.* 2018). And although one study has shown that motion does not seem to influence brain dynamics to a critical amount (Abrol *et al.* 2017), it is important to emphasize that we followed the most recent recommendations for a successful minimization of motion effects using specific data preprocessing steps (Power *et al.* 2018) (for more detailed information see Motion section).

Fifth, since complexity and the specific structure of the network are of significant importance for theories of consciousness (Baars 1988; Dehaene and Changeux 2011; Lau and Rosenthal 2011; Tononi *et al.* 2016), we investigated the dynamics of specific network properties that describe qualitative aspects of brain interaction in a quantitative way. Similar to time-varying properties, we believe that the fundamental state of a person such as whether he is conscious of his environment or not may be directly reflected by changes in the structure of the network, in contrast to the more subtle changes that occur at different levels of cognition and motor responses. We focused on only two network metrics, node strength and clustering coefficient for the following reasons: considering the small sample size and the sheer vast number of properties one can assess, we decided to assess a limited number of metrics as opposed to assessing all potential measures and then selecting the best *post hoc* or enforcing a large multiple-comparison correction. We settled on these two measures because they are among the most common metrics implemented in brain research and have been used in previous research investigating temporal dynamics (Yu *et al.* 2015) while also being appropriate measures to reflect upon the network’s ability to segregate and integrate information at the local level. Of course, other metrics have also been employed in the context of the study of consciousness [e.g. normalized characteristic path length (Monti *et al.* 2013) and modularity (Crone *et al.* 2014)]. However, since we were especially interested in the network properties of specific nodes in the basal ganglia-thalamo-cortical circuit, global measures such as modularity which cannot be calculated locally could not be applied.

Sixth, due to the acute clinical setting in the intensive care unit, subsets of our data were acquired with different MRI parameters. However, it has been shown that correlation estimates are very stable across spatial resolution (i.e. voxel size), temporal resolution (i.e. TR), and duration of data acquisition (exceeding 5 min) (Van Dijk *et al.* 2010). Furthermore, analyses also confirm that there are no significant differences in the TR between groups and sessions (*P *≥* *0.5 for all comparisons).

Seventh, because we had a specific prediction as to the pattern we ought to observe if brain dynamics are a reflection of one’s state of consciousness, we implemented our hypothesis testing as the conjunction of four independent tests (see Fig. 1). Of course, other approaches could have been applied. Specifically, a mixed-design ANOVA could have been an appropriate approach for a between-groups design with repeated measures. However, this approach would not necessarily be able to test the patterns of interest because in case of a non-significant interaction testing simple effects would not be justified. Yet, the simple effects are required to test the expected pattern. Especially given the lower degrees of freedom in an ANOVA design and the small sample size, a significant interaction is not necessarily expected. Thus, a joined test of between- and within-subjects t-test was deemed the appropriate choice for this specific analysis.

Eighth, in the context of severe brain injury behavioral scales such as the Coma Recovery Scale-Revised (Giacino and Kalmar 2006; Giacino *et al*. 2012) have been shown to be more sensitive to a patient’s state of consciousness (Schnakers *et al.* 2006) as compared to the GCS. However, due to the acute setting, the GCS is generally considered more appropriate. In the follow-up session, the vast majority of the patients had recovered to a level of behavior exceeding the Coma Recovery Scale-Revised’s and GCS’ ceiling scores, thus, making either scale not suitable for most patients. In consequence, we were not able to test differences for the distinctive subscales which would have provided more information regarding specific aspects of behavioral responsiveness.

In conclusion, we believe that it is important to reinforce a more hypothesis-driven and experimental approach in research on the state of consciousness. In addition to the common correlative and exploratory studies in this field, such an approach enables a much more justified interpretation of findings, and thus, provides further insight into the neuronal correlates of impaired consciousness. Following this approach, this is the first study attempting to provide experimental data supporting the wide-spread assumption that brain dynamics are sensitive to states of consciousness in patients after severe brain injury, and therefore, may be a promising target for future studies to identify a surrogate biomarker of consciousness based on task-free neuroimaging (Monti and Lutkenhoff 2016).

## Supplementary Material

niaa008_Supplementary_Data
