# Supplementary material for: A systematic investigation of the association between network dynamics in the human brain and the state of consciousness
Source: Neurosci Conscious. 2020 Jun 14;2020(1):niaa008. doi: 10.1093/nc/niaa008 (PMC7293819; doi:10.1093/nc/niaa008)
Supplement: niaa008_Supplementary_Data [file niaa008_supplementary_data.pdf]

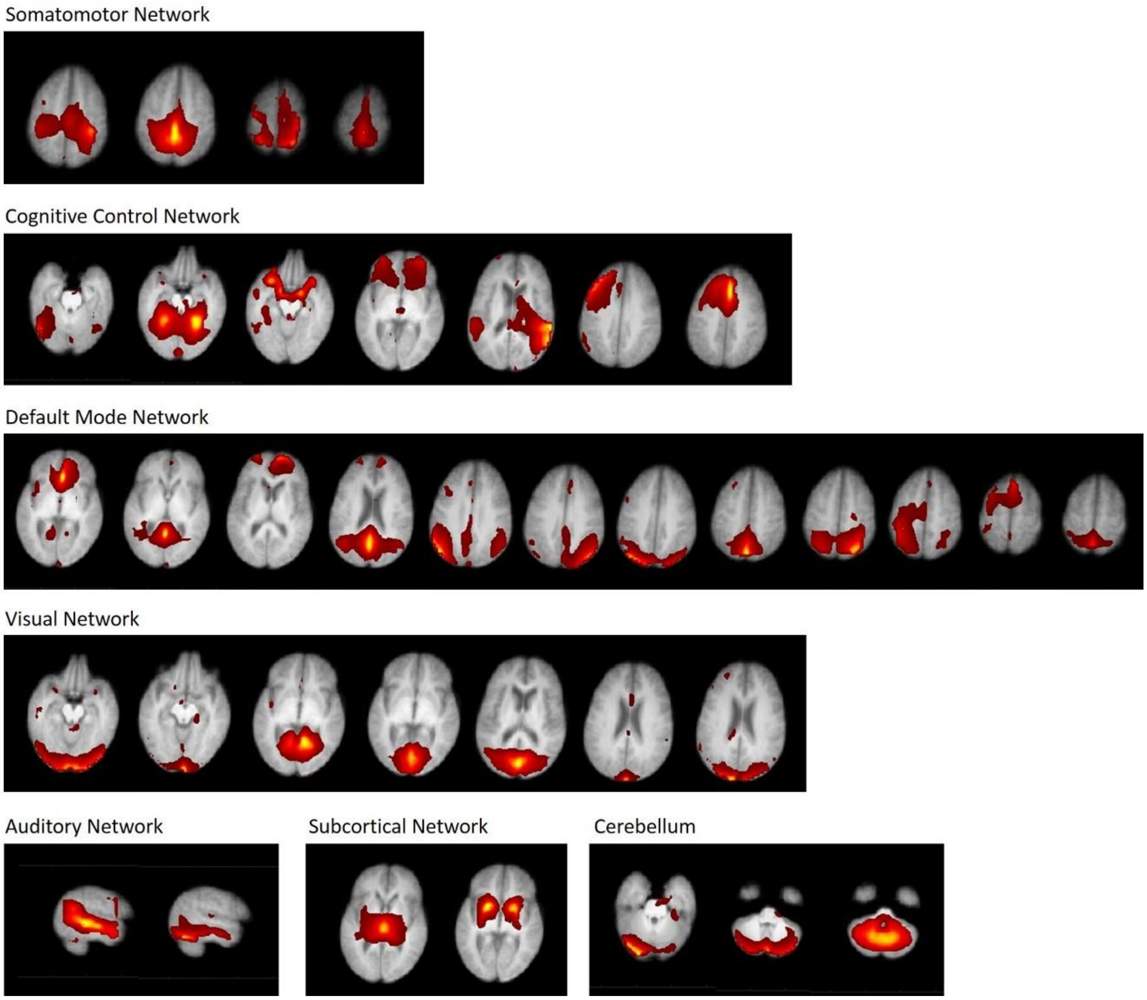

Supplementary Figure S1. Images of all included brain components using independent component analysis as a parcellation tool.

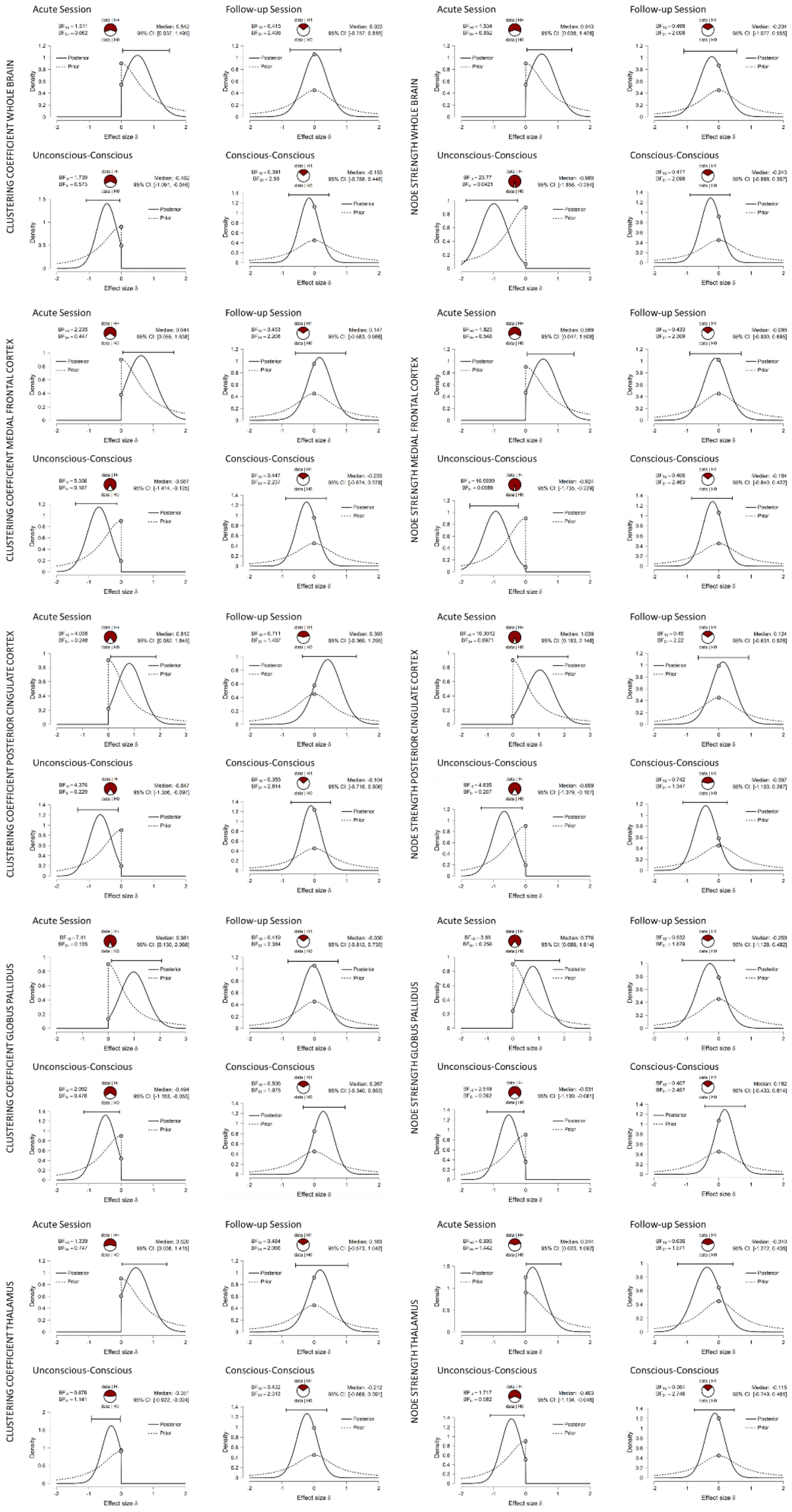
CLUSTERING COEFFICIENT WHOLE BRAIN
NODE STRENGTH WHOLE BRAIN
CLUSTERING COEFFICIENT MEDIAL FRONTAL CORTEX
NODE STRENGTH MEDIAL FRONTAL CORTEX
CLUSTERING COEFFICIENT POSTERIOR CINGULATE CORTEX
NODE STRENGTH POSTERIOR CINGULATE CORTEX
CLUSTERING COEFFICIENT GLOBUS PALLIDUS
NODE STRENGTH GLOBUS PALLIDUS
CLUSTERING COEFFICIENT THALAMUS
NODE STRENGTH THALAMUS
Acute Session
Follow-up Session
Unconscious-Conscious
Conscious-Conscious
Posterior
Prior
Density
Effect size δ

Supplementary Figure S2. Graphical representation of the prior and posterior. Bayes factor for the alternative hypothesis (BF_{10}) and for the alternative hypothesis (BF_{01}).

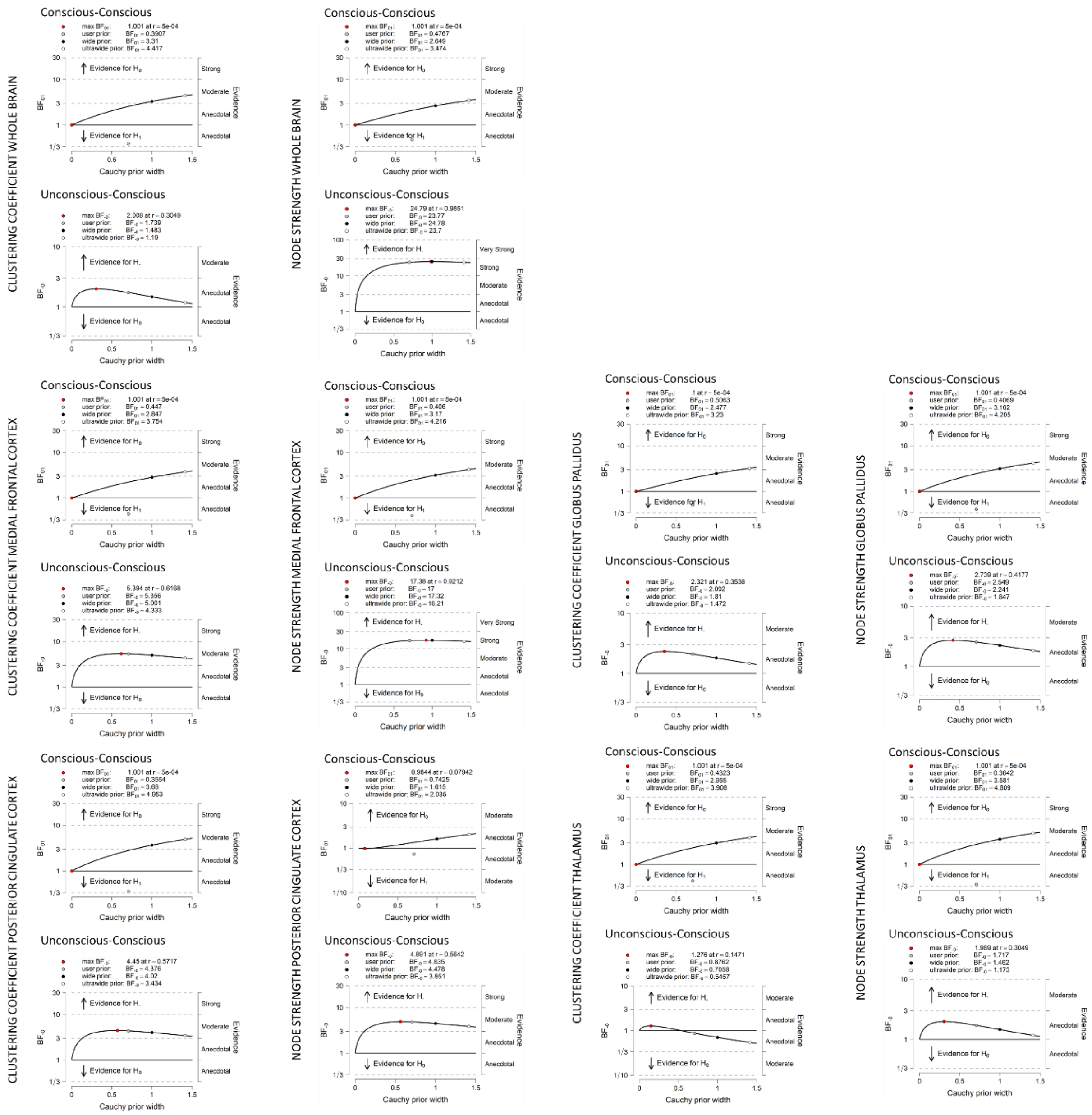

Supplementary Figure S3. Graphical representation of the Bayes factor (BF) robustness check displaying the BF for the hypothesis of interest across different widths of the Cauchy prior *r*.

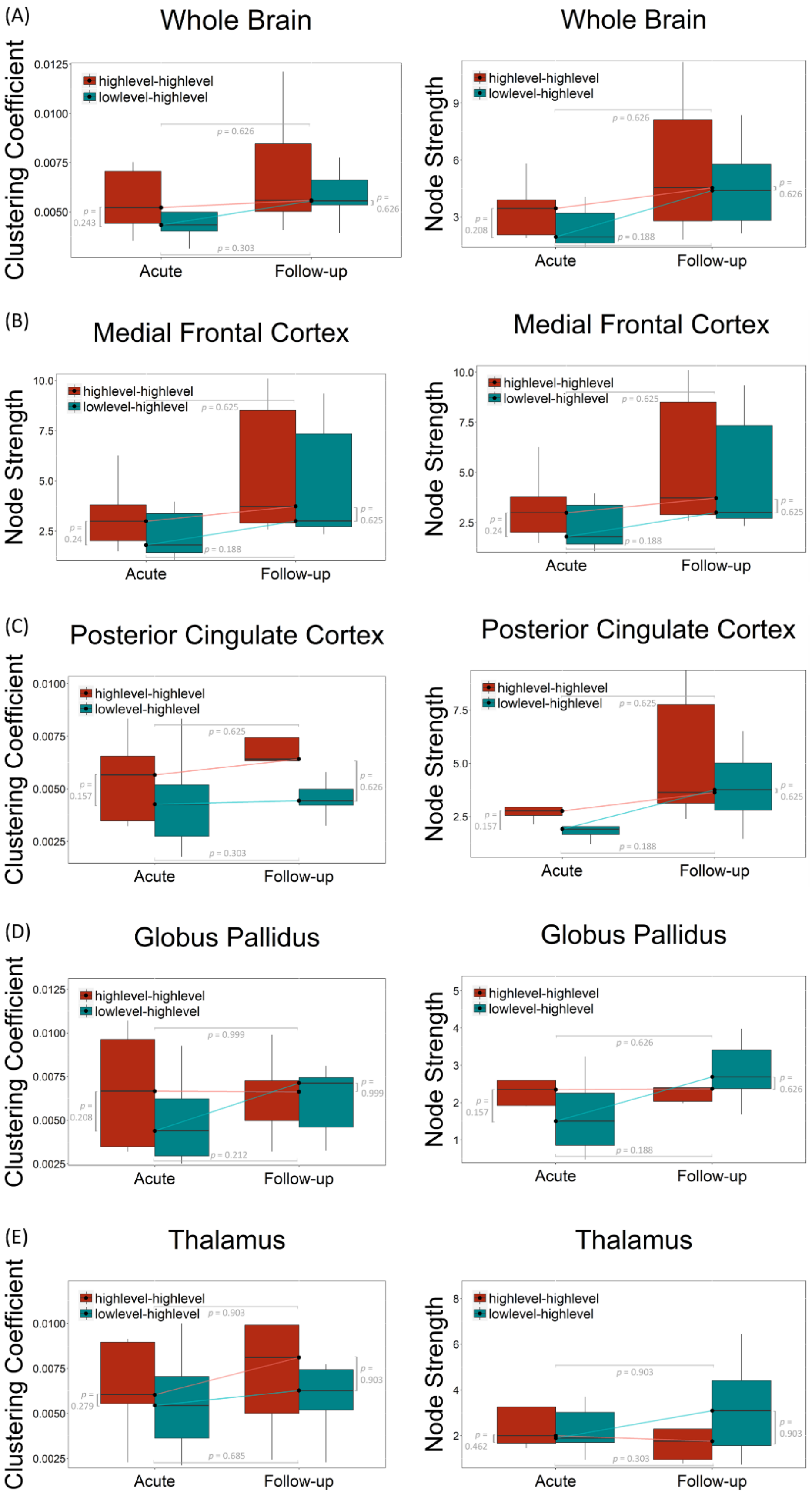
(A)
Whole Brain
Clustering Coefficient
highlevel-highlevel
lowlevel-highlevel
p = 0.626
p = 0.243
p = 0.303
Acute
Follow-up
Whole Brain
Node Strength
p = 0.208
p = 0.188
(B)
Medial Frontal Cortex
Node Strength
p = 0.625
p = 0.24
p = 0.188
(C)
Posterior Cingulate Cortex
Clustering Coefficient
p = 0.625
p = 0.157
p = 0.626
p = 0.303
(D)
Globus Pallidus
p = 0.999
p = 0.208
p = 0.212
p = 0.626
p = 0.157
p = 0.188
(E)
Thalamus
p = 0.903
p = 0.279
p = 0.685
p = 0.462
p = 0.303

Supplementary Figure S4. Pattern of differences in time-varying network properties (clustering coefficient and node strength) between low-level and high-level behavioral responsiveness. There are no significant differences between groups or sessions for the whole brain (A), the medial frontal cortex (B), the posterior cingulate cortex (C), the globus pallidus (D), and the thalamus (E). Box plots display the median, maximum and minimum value, as well as the quartiles. The *p*-values for the conscious-conscious group are displayed above, for the unconscious-conscious group below the line graphs. The *p*-values for the acute session are displayed on the left side, the *p*-values for the follow-up session are displayed on the right side. All *p*-values are FDR-corrected for multiple comparisons.

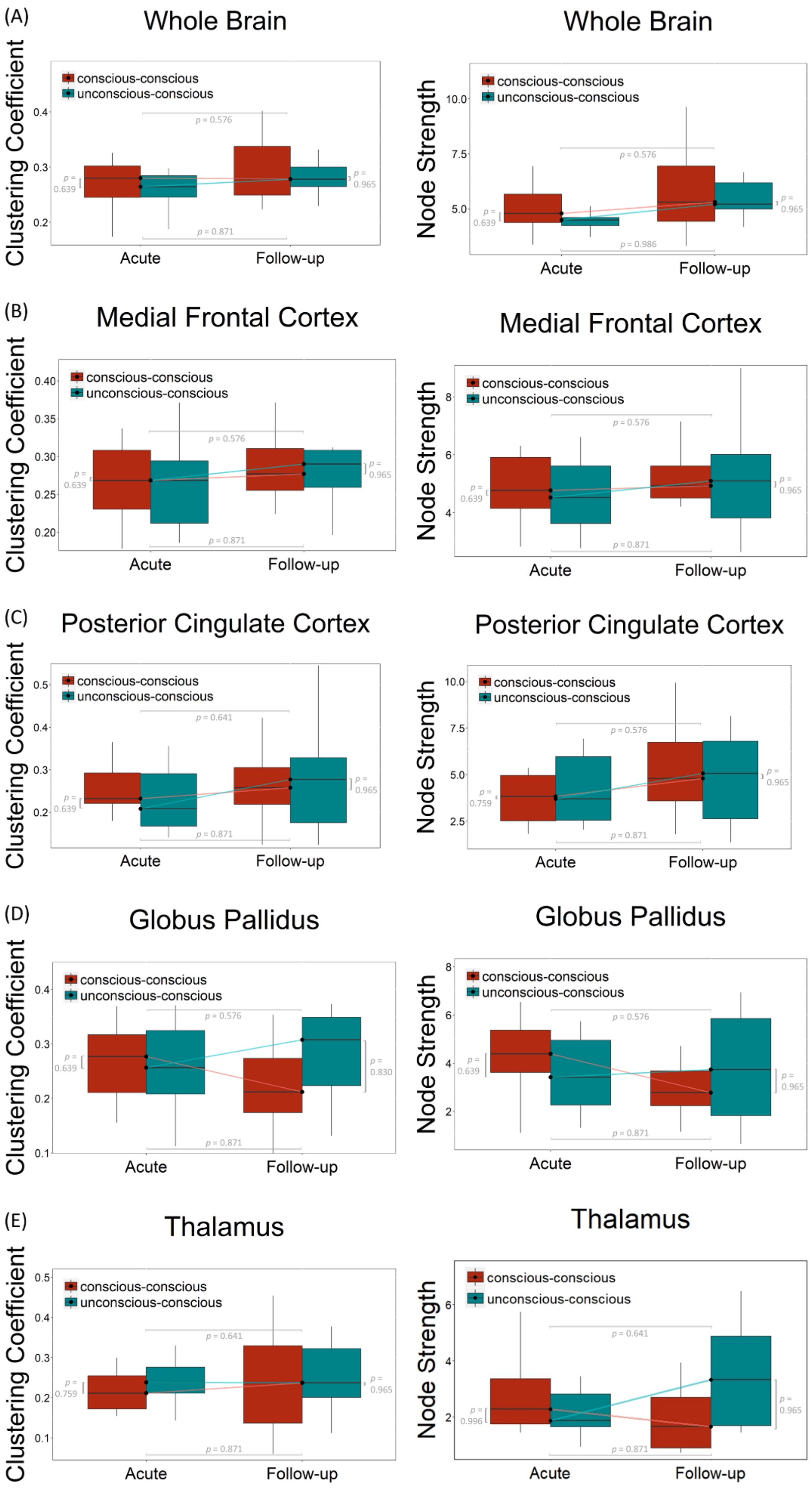
(A)
Whole Brain
Clustering Coefficient
conscious-conscious
unconscious-conscious
p = 0.576
p = 0.639
p = 0.965
p = 0.871
Acute
Follow-up
Whole Brain
Node Strength
conscious-conscious
unconscious-conscious
p = 0.576
p = 0.639
p = 0.965
p = 0.986
Acute
Follow-up
(B)
Medial Frontal Cortex
Clustering Coefficient
conscious-conscious
unconscious-conscious
p = 0.576
p = 0.639
p = 0.965
p = 0.871
Acute
Follow-up
Medial Frontal Cortex
Node Strength
conscious-conscious
unconscious-conscious
p = 0.576
p = 0.639
p = 0.965
p = 0.871
Acute
Follow-up
(C)
Posterior Cingulate Cortex
Clustering Coefficient
conscious-conscious
unconscious-conscious
p = 0.641
p = 0.639
p = 0.965
p = 0.871
Acute
Follow-up
Posterior Cingulate Cortex
Node Strength
conscious-conscious
unconscious-conscious
p = 0.576
p = 0.759
p = 0.965
p = 0.871
Acute
Follow-up
(D)
Globus Pallidus
Clustering Coefficient
conscious-conscious
unconscious-conscious
p = 0.576
p = 0.639
p = 0.830
p = 0.871
Acute
Follow-up
Globus Pallidus
Node Strength
conscious-conscious
unconscious-conscious
p = 0.576
p = 0.639
p = 0.965
p = 0.871
Acute
Follow-up
(E)
Thalamus
Clustering Coefficient
conscious-conscious
unconscious-conscious
p = 0.641
p = 0.759
p = 0.965
p = 0.871
Acute
Follow-up
Thalamus
Node Strength
conscious-conscious
unconscious-conscious
p = 0.641
p = 0.996
p = 0.965
p = 0.871
Acute
Follow-up

Supplementary Figure S5. Pattern of differences in stationary functional connectivity properties (clustering coefficient and node strength). There are no significant differences between groups or sessions for the whole brain (A), the medial frontal cortex (B), the posterior cingulate cortex (C), the globus pallidus (D), and the thalamus (E). Box plots display the median, maximum and minimum value, as well as the quartiles. The *p*-values for the conscious-conscious group are displayed above, for the unconscious-conscious group below the line graphs. The *p*-values for the acute session are displayed on the left side, the *p*-values for the follow-up session are displayed on the right side. All *p*-values are FDR-corrected for multiple comparisons.

Supplementary Table S1. Patient's information and demographics.

Patient ID	Age	Sex	Cause of Injury	GCS	Inferred GOSe	GOSe	Group
Patient 1	47	F	Blunt Trauma	11 (E:4, V:1, M:6)	3	7	conscious-conscious
Patient 2	24	M	Motorcycle Accident	5 (E:1, V:1, M:3)	2	8	unconscious-conscious
Patient 3	20	F	Car Accident	8 (E:1, V:1, M:6)	3	8	conscious-conscious
Patient 4	62	F	Bicycle Fall	7 (E:2, V:1, M:4)	2	8	unconscious-conscious
Patient 5	62	m	Fall	6 (E:1, V:1, M:4)	2	4	unconscious-conscious
Patient 6	65	m	Pedestrian Strike	8 (E:2, V:1, M:5)	3	7	conscious-conscious
Patient 7	43	m	Bicycle Strike	7 (E:2, V:1, M:4)	2	5	unconscious-conscious
Patient 8	38	m	Fall	7 (E:2, V:1, M:4)	2	8	unconscious-conscious
Patient 9	34	m	Fall	14 (E:3, V:5, M:6)	3	8	conscious-conscious
Patient 10	18	f	Motor Vehicle Accident	6 (E: 1, V:1, M:4)	2	7	unconscious-conscious
Patient 11	23	f	Pedestrian Strike	7 (E:3, M:3, V:1)	2	7	unconscious-conscious
Patient 12	21	f	Fall	15 (E:4, V:5, M:6)	5	8	conscious-conscious
Patient 13	25	m	Pedestrian Strike	3 (E:1, V:1, M:1)	2	8	unconscious-conscious
Patient 14	23	f	Motorcycle Accident	3 (E:1, V:1, M:1)	2	3	unconscious-conscious
Patient 15	76	f	Fall	15 (E:4, V:5, M:6)	3	7	conscious-conscious
Patient 16	43	m	Fall from Height	8 (E:2, V:1, M:5)	3	4	conscious-conscious
Patient 17	39	m	Pedestrian Strike	6 (E:1, V:1, M:4)	2	3	unconscious-conscious
Patient 18	28	m	Motorcycle Accident	10 (E:4, V:1, M:5)	3	4	conscious-conscious

GCS=Glasgow Coma Scale; GOSe=Glasgow Outcome Scale extended; E=Eye subscore; V=verbal subscore; M=motor subscore;

Supplementary Table S2. Patient's fMRI acquisition parameters.

Patient ID	Matrix Size	Voxel Size	Acquisition Order	TR	TE	Timepoints
Patient 1 acute	64x64	3.4375x3.4375x3	Sequential decreasing	3	25	200
Patient 1 follow-up	64x64	3.4375x3.4375x3	Sequential decreasing	3	25	200
Patient 2 acute	64x64	3.4375x3.4375x3	Sequential decreasing	3	25	200
Patient 2 follow-up	64x64	3.4375x3.4375x3	Sequential decreasing	3.19	25	200
Patient 3 acute	64x64	3.4375x3.4375x3	Sequential decreasing	3	25	200
Patient 3 follow-up	64x64	3.4375x3.4375x3	Sequential decreasing	3	25	200
Patient 4 acute	64x64	3.4375x3.4375x3	Sequential decreasing	3	25	200
Patient 4 follow-up	64x64	3.4375x3.4375x3	Sequential decreasing	3	25	200
Patient 5 acute	64x64	3.5x3.5x3	Alternating increasing	3	25	200
Patient 5 follow-up	64x64	3.4375x3.4375x3	Sequential decreasing	3	25	200
Patient 6 acute	64x64	3.4375x3.4375x3	Sequential decreasing	3	25	200
Patient 6 follow-up	64x64	3.4375x3.4375x3	Sequential decreasing	3.06	25	200
Patient 7 acute	64x64	3.4375x3.4375x3	Sequential decreasing	3	25	200
Patient 7 follow-up	64x64	3.4375x3.4375x3	Sequential decreasing	3	25	200
Patient 8 acute	64x64	3.4375x3.4375x3	Sequential decreasing	3	25	200
Patient 8 follow-up	64x64	3.4375x3.4375x3	Sequential decreasing	3.14	25	200
Patient 9 acute	64x64	3.4375x3.4375x3	Sequential decreasing	3	25	200
Patient 9 follow-up	64x64	3.4375x3.4375x3	Sequential decreasing	3	25	200
Patient 10 acute	64x64	3.4375x3.4375x3	Sequential decreasing	3	25	200
Patient 10 follow-up	64x64	3.4375x3.4375x3	Sequential decreasing	3	25	200
Patient 11 acute	64x64	3.4375x3.4375x3	Sequential decreasing	3	25	200
Patient 11 follow-up	64x64	3.4375x3.4375x3	Sequential decreasing	3	25	200
Patient 12 acute	64x64	3.4375x3.4375x3	Sequential decreasing	3	25	200
Patient 12 follow-up	64x64	3.4375x3.4375x4.25	Sequential decreasing	2	25	200
Patient 13 acute	64x64	3.4375x3.4375x3	Sequential decreasing	3	25	200
Patient 13 follow-up	64x64	3.4375x3.4375x4.25	Sequential decreasing	2.06	25	200
Patient 14 acute	64x64	3.4375x3.4375x3	Sequential decreasing	3	25	200
Patient 14 follow-up	64x64	3.4375x3.4375x4.25	Sequential decreasing	2.06	25	200
Patient 15 acute	64x64	3.4375x3.4375x3	Sequential decreasing	3	25	200
Patient 15 follow-up	64x64	3.4375x3.4375x4.25	Sequential decreasing	2.06	25	200
Patient 16 acute	64x64	3x3x3.99	Alternating decreasing	2	25	200
Patient 16 follow-up	64x64	3.4375x3.4375x4.25	Sequential decreasing	2.06	25	200
Patient 17 acute	64x64	3.4375x3.4375x4.2	Sequential decreasing	2.06	25	200
Patient 17 follow-up	64x64	3.4375x3.4375x4.25	Sequential decreasing	2.06	25	200
Patient 18 acute	64x64	3.4375x3.4375x4.25	Sequential decreasing	2	25	200
Patient 18 follow-up	64x64	3.4375x3.4375x4.25	Sequential decreasing	2.06	25	200

Supplementary Table S3. Results of the permutation t-tests for each comparison.

**Dynamic network interaction**		
**Brain Area**	**Comparison**	***p***
Whole brain clustering coefficient	Acute between groups	0.064
	Unconscious-conscious within group	0.082
	Follow-up between groups	0.452
	Conscious-conscious within group	0.688
Whole brain node strength	Acute between groups	0.037
	Unconscious-conscious within group	0.006
	Follow-up between groups	0.595
	Conscious-conscious within group	0.595
MFC clustering coefficient	Acute between groups	0.009
	Unconscious-conscious within group	0.033
	Follow-up between groups	0.342
	Conscious-conscious within group	0.547
MFC node strength	Acute between groups	0.036
	Unconscious-conscious within group	0.006
	Follow-up between groups	0.663
	Conscious-conscious within group	0.342
PCC clustering coefficient	Acute between groups	0.037
	Unconscious-conscious within group	0.017
	Follow-up between groups	0.773
	Conscious-conscious within group	0.114
PCC node strength	Acute between groups	0.039
	Unconscious-conscious within group	0.036
	Follow-up between groups	0.188
	Conscious-conscious within group	0.253
GP clustering coefficient	Acute between groups	0.004
	Unconscious-conscious within group	0.23
	Follow-up between groups	0.56
	Conscious-conscious within group	0.359
GP node strength	Acute between groups	0.022
	Unconscious-conscious within group	0.29
	Follow-up between groups	0.82
	Conscious-conscious within group	0.531
Thalamus clustering coefficient	Acute between groups	0.064
	Unconscious-conscious within group	0.269
	Follow-up between groups	0.225
	Conscious-conscious within group	0.664
Thalamus node strength	Acute between groups	0.25
	Unconscious-conscious within group	0.117
	Follow-up between groups	0.524
	Conscious-conscious within group	0.922
**Stationary network interaction**		
**Brain Area**	**Comparison**	***p***
Whole brain clustering coefficient	Acute between groups	0.321
	Low level-high level within group	0.26
	Follow-up between groups	0.166
	High level-high level within group	0.313
Whole brain node strength	Acute between groups	0.176
	Low level-high level within group	0.036
	Follow-up between groups	0.208

	High level-high level within group	0.25
MFC clustering coefficient	Acute between groups	0.156
	Low level-high level within group	0.062
	Follow-up between groups	0.192
	High level-high level within group	0.5
MFC node strength	Acute between groups	0.238
	Low level-high level within group	0.061
	Follow-up between groups	0.229
	High level-high level within group	0.125
PCC clustering coefficient	Acute between groups	0.134
	Low level-high level within group	0.336
	Follow-up between groups	0.038
	High level-high level within group	0.375
PCC node strength	Acute between groups	0.153
	Low level-high level within group	0.053
	Follow-up between groups	0.122
	High level-high level within group	0.125
GP clustering coefficient	Acute between groups	0.317
	Low level-high level within group	0.317
	Follow-up between groups	0.932
	High level-high level within group	0.313
GP node strength	Acute between groups	0.133
	Low level-high level within group	0.169
	Follow-up between groups	0.767
	High level-high level within group	0.875
Thalamus clustering coefficient	Acute between groups	0.573
	Low level-high level within group	0.945
	Follow-up between groups	0.276
	High level-high level within group	0.813
Thalamus node strength	Acute between groups	0.28
	Low level-high level within group	0.204
	Follow-up between groups	0.307
	High level-high level within group	0.938
**Stationary network interaction**		
**Brain Area**	**Comparison**	***p***
Whole brain clustering coefficient	Acute between groups	0.239
	Unconscious-conscious within group	0.305
	Follow-up between groups	0.434
	Conscious-conscious within group	0.281
Whole brain node strength	Acute between groups	0.074
	Unconscious-conscious within group	0.029
	Follow-up between groups	0.553
	Conscious-conscious within group	0.461
MFC clustering coefficient	Acute between groups	0.48
	Unconscious-conscious within group	0.391
	Follow-up between groups	0.54
	Conscious-conscious within group	0.172
MFC node strength	Acute between groups	0.36
	Unconscious-conscious within group	0.45
	Follow-up between groups	0.53
	Conscious-conscious within group	0.461
PCC clustering coefficient	Acute between groups	0.224
	Unconscious-conscious within group	0.438

	Follow-up between groups	0.47
	Conscious-conscious within group	0.5
PCC node strength	Acute between groups	0.69
	Unconscious-conscious within group	0.387
	Follow-up between groups	0.56
	Conscious-conscious within group	0.211
GP clustering coefficient	Acute between groups	0.417
	Unconscious-conscious within group	0.443
	Follow-up between groups	0.934
	Conscious-conscious within group	0.219
GP node strength	Acute between groups	0.175
	Unconscious-conscious within group	0.822
	Follow-up between groups	0.763
	Conscious-conscious within group	0.133
Thalamus clustering coefficient	Acute between groups	0.604
	Unconscious-conscious within group	0.635
	Follow-up between groups	0.722
	Conscious-conscious within group	0.953
Thalamus node strength	Acute between groups	0.994
	Unconscious-conscious within group	0.856
	Follow-up between groups	0.55
	Conscious-conscious within group	0.336

Supplementary Table S4. Effect sizes for stationary network interaction.

Brain Area	Comparison	*r*	Effect size
Whole brain clustering coefficient	Acute between groups	0.2	Small
	Unconscious-conscious within group	0.309	Medium
	Follow-up between groups	0.025	Negligible
	Conscious-conscious within group	0.556	Large
Whole brain node strength	Acute between groups	0.714*	Medium
	Unconscious-conscious within group	0.745	Large
	Follow-up between groups	0.050	Negligible
	Conscious-conscious within group	0.333	Medium
MFC clustering coefficient	Acute between groups	0.050	Negligible
	Unconscious-conscious within group	0.273	Small
	Follow-up between groups	0.063	Negligible
	Conscious-conscious within group	0.5	Large
MFC node strength	Acute between groups	0.1	Small
	Unconscious-conscious within group	0.2	Small
	Follow-up between groups	0.025	Negligible
	Conscious-conscious within group	0.333	Medium
PCC clustering coefficient	Acute between groups	0.325	Medium
	Unconscious-conscious within group	0.273	Small
	Follow-up between groups	0.075	Negligible
	Conscious-conscious within group	0.222	Small
PCC node strength	Acute between groups	0.125	Small
	Unconscious-conscious within group	0.127	Small
	Follow-up between groups	0.025	Negligible
	Conscious-conscious within group	0.389	Medium
GP clustering coefficient	Acute between groups	0.075	Negligible
	Unconscious-conscious within group	0.164	Small
	Follow-up between groups	0.5	Large
	Conscious-conscious within group	0.5	Large
GP node strength	Acute between groups	0.225	Small
	Unconscious-conscious within group	0.091	Negligible
	Follow-up between groups	0.225	Small
	Conscious-conscious within group	0.611	Large
Thalamus clustering coefficient	Acute between groups	0.125	Small
	Unconscious-conscious within group	0.055	Negligible
	Follow-up between groups	0.15	Small
	Conscious-conscious within group	0.222	Small
Thalamus node strength	Acute between groups	0.7	Large
	Unconscious-conscious within group	0.309	Medium
	Follow-up between groups	0.15	Small
	Conscious-conscious within group	0.333	Medium

r represents the rank biserial correlation coefficient for the effect size; * indicates that the Cohen's d for effect size has been used due to unequal variances.
